# People with intellectual and sensory disabilities can independently start and perform functional daily activities with the support of simple technology

**DOI:** 10.1371/journal.pone.0269793

**Published:** 2022-06-13

**Authors:** Giulio E. Lancioni, Nirbhay N. Singh, Mark F. O’Reilly, Jeff Sigafoos, Gloria Alberti, Valentina Del Gaudio, Chiara Abbatantuono, Paolo Taurisano, Lorenzo Desideri

**Affiliations:** 1 Department of Neuroscience and Sense Organs, University of Bari, Bari, Italy; 2 Department of Psychiatry, Augusta University, Augusta, GA, United States of America; 3 College of Education, University of Texas at Austin, Austin, GA, United States of America; 4 School of Education, Victoria University of Wellington, Wellington, New Zealand; 5 Lega F. D’Oro Research Center, Osimo, Italy; University of Perugia: Universita degli Studi di Perugia, ITALY

## Abstract

**Objectives:**

The study assessed a smartphone-based technology system, which was designed to enable six participants with intellectual disability and sensory impairment to start and carry out functional activities through the use of reminders and verbal or pictorial instructions.

**Methods:**

The technology system involved a Samsung Galaxy A22 with Android 11 operating system and four Philips Hue indoor motion sensors. Three to five activities were scheduled per day. At the time at which an activity was due, the system provided the participant with a reminder followed by the verbal or pictorial instruction for the initial part of the first response (e.g., “Go to the bathroom and take the dirty towels”). The instruction would be available (repeated) until the participant responded to it and, in so doing, activated a sensor. Sensor activation caused the presentation of the instruction for the second part of the same (first) response (e.g., “Put the towels in the laundry machine”). The same process occurred for each of the responses involved in the activity. The system was introduced according to nonconcurrent multiple baseline designs across participants.

**Results:**

During baseline, the mean percentage of activities the participants started independently was below 7; the mean frequency of correct responses per activity was below 0.5 (out of a maximum possible of 8). During the intervention (i.e., with the support of the technology system), the mean percentage and mean frequency values increased to nearly 100 and 8, respectively.

**Conclusions:**

The data suggest that the aforementioned technology system may enable people with intellectual disability and sensory impairment to start and carry out functional activities independent of staff.

## Introduction

People with intellectual disabilities or combinations of intellectual and sensory or motor disabilities may need specific staff or caregiver’s support to initiate and complete activities with multiple steps, such as washing dishes, putting away clothes, or assembling objects [1–3]. In fact, they (a) may not know when those activities are due and/or lack any initiative to start them and (b) may not remember the steps included in the activities and the order in which those steps are to be carried out [4–8]. Their dependence on external support to be constructively engaged in functional activity may require a level of supervision that many daily contexts might not be able to afford. This may often result in reduced levels of engagement, with negative implications for the development or strengthening of functional occupation and self-determination skills, as well as for the social image [9–11].

One way to counter the aforementioned situation involves the use of technology-aided programs designed to provide verbal or pictorial (i.e., picture- or video-based) step instructions enabling the participants to carry out the activities included in the program [6,12–16]. Four different types of programs have been developed. The first type of program involves the use of devices such as computers or tablets presenting the step instructions (one at a time) in relation to the participants’ activation of those devices (e.g., through a response such as touching the tablet or computer screen) [6,12,17–19]. The second type of program differs from the first in that it uses a computer, tablet or smartphone to automatically present step instructions at preset time intervals [14,15,20]. The intervals are arranged in advance by staff with the view of facilitating the participants’ performance, that is, of freeing the participants from the need of (and possible errors in) activating the devices for each activity step. The third type of program, which can rely on the use of a smartphone or tablet, is designed not only to provide instructions for the activity steps at preset intervals but also to cue/alert the participants when any activity is to be started so that the participants can initiate the activities on their own at the right time [14,15,21]. The fourth type of program differs from the second because the instructions are presented automatically to the participants as the participants reach the locations where the objects for the activity steps are to be used [16,22]. To detect the participants’ arrival at the target locations, specific environmental sensors (e.g., dance pads) are employed.

All the different types of programs have shown their usefulness in helping participants with intellectual and other disabilities carry out complex (multistep) activities with satisfactory levels of completeness/accuracy [6,13,15,22]. Obviously, the third and fourth types of programs might be considered more advanced than the first two in terms of the level and quality of support they offer to the participants. Combining the advantageous characteristics of the last two types of programs could lead to a new (upgraded) program that would automatically (a) present the activity instructions in functional connection with the participants’ performance and (b) provide timely cues for the start of the activities.

The purpose of this study was to set up and assess such a new program. Within this program, presenting the instructions in functional connection with the participant’s performance entailed (a) delivering the instructions at the time the participant was ready to perform the related/intended actions and (b) making the instructions available (i.e., repeating or extending them) until the participant was ready to perform the related/intended actions. In contrast to what was done in previous studies [16,22], the instructions scheduled for the participants’ performance were not limited to a single activity, but covered various activities, which involved several destinations/target areas. The technology system was meant to be suitable for planning flexible activities (i.e., activities that could be easily modified across different days in terms of responses/steps included, so that they could better suit the context and the participants’ conditions). The technology system was based on the use of a smartphone and motion sensors, which for some participants were connected to a Bluetooth mini speaker. Six participants with intellectual disability and visual or auditory impairments were included in the study.

## Method

### Participants

Table 1 identifies the six participants by their pseudonyms and reports their chronological age, their visual and auditory conditions, and the age equivalents for their daily living skills on the second edition of the Vineland Adaptive Behavior Scales [23,24]. The participants’ chronological age ranged from 35 to 61 years. Two participants had typical vision (Chelsea and Nancy); three participants (Liz, Mary, and George) had a functional residual vision that allowed them to discriminate objects as well as pictures of objects and locations; and one participant (Andy) had only perception of light and shadows. Regarding the auditory condition, four participants (Liz, Andy, Mary, and George) had typical hearing while the other two participants (Chelsea and Nancy) had severe hearing loss. One participant (George) also had motor impairment and used an electric wheelchair for all his indoor travel. The Vineland age equivalents for their daily living skills (personal sub-domain) varied between 4 years and 1 month (Liz and George) and 5 years and 6 months (Nancy). All participants attended rehabilitation and care centers. The psychological services of those centers reported the participants’ intellectual disability to range within the moderate spectrum. Available estimates suggest that individuals with moderate intellectual disability represent about 10% of the cases of this clinical population and need some assistance in several areas of their daily life including engagement in functional activities [25].

**Table 1:** Participants’ pseudonyms, chronological age, visual and auditory conditions, and Vineland age equivalents for daily living skills (personal sub-domain).

Participants (pseudonyms)	Chronological age (years)	Visual and auditory conditions	Vineland age equivalents ^1^^,^^2^
Liz	46	Functional residual vision; Typical hearing	4; 1
Andy	52	Perception of light and shadows; Typical hearing	5; 3
Mary	46	Functional residual vision; Typical hearing	4; 4
George	35	Functional residual vision; Typical hearing	4; 1
Chelsea	59	Typical vision; Severe hearing loss	5; 3
Nancy	61	Typical vision; Severe hearing loss	5; 6

^1^The age equivalents are based on the Italian standardization of the Vineland scales [23].

^2^The Vineland age equivalents are reported in years (number before the semicolon) and months (number after the semicolon).

The participants could independently orient and travel within their living and occupational contexts. They also were able to respond to simple verbal or pictorial instructions concerning daily activity steps both when presented by staff and when presented by a smartphone or other technology device (i.e., could profitably use the support of the second or fourth types of programs mentioned above). However, the participants were not able to carry out multistep activities independently (i.e., without instructions) and did not typically start activities on their own initiative.

### Ethical approval and informed consent

All participants had been informed verbally and through demonstrations as to how the smartphone-based system used in this study worked, and had expressed their willingness to be involved in the study and carry out daily activities with the support of the system. In light of the participants’ intellectual disability level, this willingness was considered to be a reliable indicator of their consent for the study. However, given their inability to read and sign a consent form, their legal representatives were asked to fulfill those requirements on their behalf. The study complied with the 1964 Helsinki declaration and its later amendments and was approved (including the aforementioned consent procedures) by an institutional Ethics Committee.

### Procedures

#### Setting, activities, instructions, and research assistants

The study was carried out in the centers that the participants attended. The activities were to be performed within the participants’ daily contexts (living and occupational facilities). The participants could easily move inside those contexts and find the rooms/areas and objects involved in the activities. Activities consisted of combinations of functional responses (steps), that is, responses that were known and meaningful to the participants and useful within the context. Between 50 and 71 functional responses were identified/available for each participant. Various combinations/groups of eight such responses (which could change across days) were considered to represent viable activities, that is, multifaceted forms of occupation relevant for the participant and convenient and beneficial for the context. Between three and five activities were scheduled per day over the morning, the afternoon or the entire day, typically 3 to 5 days a week. Table 2 provides two combinations of eight responses considered to represent two viable activities.

**Table 2:** Two combinations of eight responses considered to represent two viable activities.

**First Combination (Activity 1)**
• Go to the bathroom, take the dirty towels, and put them in the laundry machine.
• Go to the cabinet store, take the toilet paper, and bring it to the bathroom.
• Go to the laundry area, take your clean shirt, and put it in your closet.
• Go to the kitchen, take the dishcloths, and put them in the laundry machine.
• Go to the cabinet store, take a water bottle, and bring it to the kitchen.
• Go to the bathroom, take your razor, and put it in your closet.
• Go to the kitchen, take the tablecloth, and put it on the table.
• Go to the laundry area, take the clean dishcloths, and bring them to the kitchen.
**Second Combination (Activity 2)**
• Go to the kitchen, take the watering can, and water the plant in the red vase.
• Go to the bathroom, take the dirty clothes, and put them in the laundry machine.
• Go to the cabinet store, take the coffee pads, and put them on the kitchen table.
• Go to the kitchen, take the newspapers, and put them in the paper trashcan.
• Go to the laundry area, take your clean pants, and put them in your closet.
• Go to the kitchen, take the dishes on the table, and put them in the sink.
• Go to the cabinet store, take the paper towels, and put them on the kitchen table.
• Go to the laundry area, take clean towels, and bring them to the bathroom.

The instructions for the activities were verbal (with the addition of miniature objects during baseline phases) for Liz, Andy, and Mary and pictorial for the other three participants (i.e., George, Chelsea, and Nancy). During baseline phases, the research assistants gave instructions for (described) each activity to be carried out in a global manner (i.e., with a few phrases and objects or a drawing). During the intervention phase, the smartphone provided two verbal or pictorial instructions for each response of the activity the participants were to perform (e.g., “Go to the cabinet store and take paper towels” and “Put the paper towels on the kitchen table”). The second instruction was presented only after the participants had responded to the first one. Research assistants, who carried out the sessions and recorded the participants’ data, had experience with the application of technology-aided interventions with people with intellectual and other disabilities and also with data collection.

#### Technology system

The technology system used during the intervention phase of the study involved (a) a Samsung Galaxy A22 with Android 11 operating system that was equipped with Amazon Alexa, MacroDroid, and Philips Hue applications, (b) four Philips Hue indoor motion sensors, (c) a Philips Hue Bridge and Philips Hue smart bulb working via Bluetooth, (d) a 4G Long-Term Evolution Wi-Fi router, and (e) a Bluetooth mini speaker. The mini speaker was only used for the participants who received verbal instructions. The Philips Hue Bridge, smart bulb and application, and the router served to support the functioning of the Philips Hue sensors.

The sensors were box-like devices with a 5.5-cm side and 3.5-cm height. One sensor was placed in each of the four areas (e.g., kitchen area, bathroom, laundry area, and store cabinet) where the participants were to collect objects to perform the responses for the single activities of the day. Sensor activation (caused by the participant’s arrival at a specific area) was detected through the Amazon Alexa application and transmitted to the smartphone via the MacroDroid. In connection with this, the smartphone would (a) shelve the first instruction being delivered for the response that the participant was performing and (b) present the second instruction for the completion of such response (see below in this section). The verbal instructions were written in the MacroDroid, uttered by the smartphone through the vocal synthesis function, and delivered to the participants via the mini speaker that they carried with them. The pictorial instructions were in a folder of the smartphone directly accessed by MacroDroid, which regulated their presentation on the participants’ smartphone screen.

The research assistant would configure the smartphone with the activities the participant had to carry out during the day. This involved (a) setting the time at which each activity was to be started and (b) selecting the verbal or pictorial instructions for the responses the activity had to involve (i.e., from the list of responses available for the participant). Participants relying on verbal instructions only needed to have the mini speaker with them. Participants relying on pictorial instructions needed to have the smartphone with them.

At the time at which an activity was due, the system provided the participant with a verbal reminder about it, or vibration and light flashes from the smartphone, followed by the first instruction for the initial response of the activity. For example, the instruction could be the verbal message: “Go to the bathroom and take the dirty towels” or a picture showing dirty towels in the bathroom being removed. The verbal instruction would be repeated at programmable intervals, while the pictorial instruction remained on the smartphone screen, until the participant reached the bathroom and, in so doing, activated the sensor available in that area. Sensor activation was followed (after about 2 s) by the presentation of the instruction concerning the second part of the same (first) response. Such instruction could be the verbal phrase “Put the towels in the laundry machine” or the picture showing the towels in the laundry machine. After a time estimated to be sufficient for bringing the towels to the laundry machine (e.g., 20–30 s), the system presented the initial instruction for the second response of the activity (e.g., the verbal phrase “Go to the cabinet store and take the toilet paper” or the picture of the cabinet store and toilet paper). The phrase would be repeated at programmable intervals while the picture remained on the smartphone screen until the participant reached the cabinet store and activated the sensor available there. Sensor activation led to the delivery of the instruction for the second part of the response (e.g., “Bring the paper to the bathroom”). The instruction process for each of the following responses of the activity was as that described above.

Following the last instruction for the last response of the activity, the system presented a phrase such as “Well done, now you can return to your desk” or a picture conveying the same message. Following the last activity of the day, the system presented the aforementioned phrase/message and thereafter a preferred song or video (e.g., comedy, family event or sport).

#### Experimental conditions

Each of the two groups of participants (i.e., the three participants relying on verbal instructions and the three participants relying on pictorial instructions) was exposed to the use of the technology system according to a single-case research design, that is, a nonconcurrent multiple baseline design across participants [26,27]. Specifically, the participants started with two baseline phases (i.e., without the technology system). Within each of these phases, different (increasing) numbers of activities were available for the members of every group, in line with the design requirements. Thereafter, the participants entered the intervention phase, which involved the use of the technology system.

Video recordings of the participants during their performance of the activities scheduled were regularly accessible to a study coordinator who would view them and provide feedback to the research assistants about the way they implemented the experimental (baseline or intervention) conditions. This feedback was specifically aimed at ensuring that those conditions were implemented correctly, and thus at guaranteeing procedural fidelity [28].

#### Baseline I

This phase served to determine whether the participants would independently start the activities scheduled for them. Three or four activities were scheduled per day. At the beginning of the day (when the participant was sitting at a desk with conventional/occupational material such as kitchen utensils or objects to be assembled), the research assistant presented all the activities to be carried out during the day (a) verbally and through miniature objects for Liz, Andy, and Mary and (b) using a sheet of paper with drawings concerning those activities for George, Chelsea, and Nancy. For each of the activities presented verbally and through miniature objects, the research assistant provided the participant with global verbal instructions (e.g., you supply the bathroom, arrange the kitchen and take care of the plants) and also gave the participant known miniature objects representing the bathroom, the kitchen and plants (as extra, tangible activity cues). For each of the activities presented pictorially, the research assistant provided the participant with a drawing that showed three of the areas/objects that were to be used (e.g., bathroom, laundry machine, and kitchen). An activity was rated as “started independently” if the participant carried out at least one of the responses included in that activity. If the participant did not independently start any activity within one hour from receiving the instructions, a negative score was given for all the activities scheduled.

#### Baseline II

During this phase, the activities were presented one at a time. After presenting an activity (i.e., in the same way as in Baseline I), the research assistant asked the participant to carry it out. The research assistant would not intervene if the participant correctly performed responses that were included in the activity. Yet, the research assistant intervened by providing verbal or pictorial instructions (comparable to those used by the system; see Technology system) to support a response included in the activity (a) after the participant had performed a response that was not included in the activity and (b) after a period of passivity of about 40 s. This process continued until all the responses included in the activity were carried out.

#### Intervention

During the intervention, the technology system was used. At the beginning of the day, the participant was provided with the mini speaker (Liz, Andy, and Mary) or the smartphone (George, Chelsea, and Nancy). The system worked as described in the Technology system section. That is, for each of the three to five activities scheduled for the day, the system provided a reminder when the activity was due and followed the reminder with the delivery of instructions for every single response included in the activity. During the initial 15 activities, the research assistant would intervene with prompts/guidance if the participant showed any problem or hesitation/delay in using the system’s reminders and response instructions appropriately. Following those initial activities, the research assistant did no longer intervene except if the participant asked for help.

### Measures of participants’ performance

The measures recorded were: (a) activities started independently, (b) activity responses performed correctly, and (c) time required for the completion of each activity. Activities started independently were those initiated by the participants on their own (a) following the research assistants’ presentation of (global instructions about) the activities they were supposed to carry out for the day (Baseline I) or (b) after the reminder of the smartphone (Intervention). Correct responses for an activity were those that belonged to that activity, matched the description available for them, and were performed by the participants independently (i.e., without any research assistant’s instructions or prompt/guidance). Recording of the first two measures was carried out by the research assistants, who were also responsible for implementing the procedural conditions in the different phases of the study. Recording of the last measure was carried out via the smartphone during the intervention phase. Interrater agreement on the first two measures was checked on at least 20% of the activities of each participant with a reliability observer joining the research assistant in recording the data. Agreement for each measure was computed by dividing the number of activities on which both research assistant and reliability observer reported the same scoring (i.e., activity start scoring or response scoring) by the total number of activities checked and multiplying by 100%. The percentages of agreement were 95% or higher on both measures for each participant.

### Data analysis

The participants’ data for activities started independently and correct responses were reported in graphic form. In order to simplify the graphic display, the data were summarized over blocks of activities. Consequently, the data points appearing in the graphs represent mean percentages of activities started independently and mean frequencies of correct responses (see Results). The Kolmogorov-Smirnov test [29] was to be used for analyzing the differences between the data of Baseline I and Baseline II and the data of the Intervention phase for the participants who showed data overlaps between those phases.

## Results

**Fig 1:**
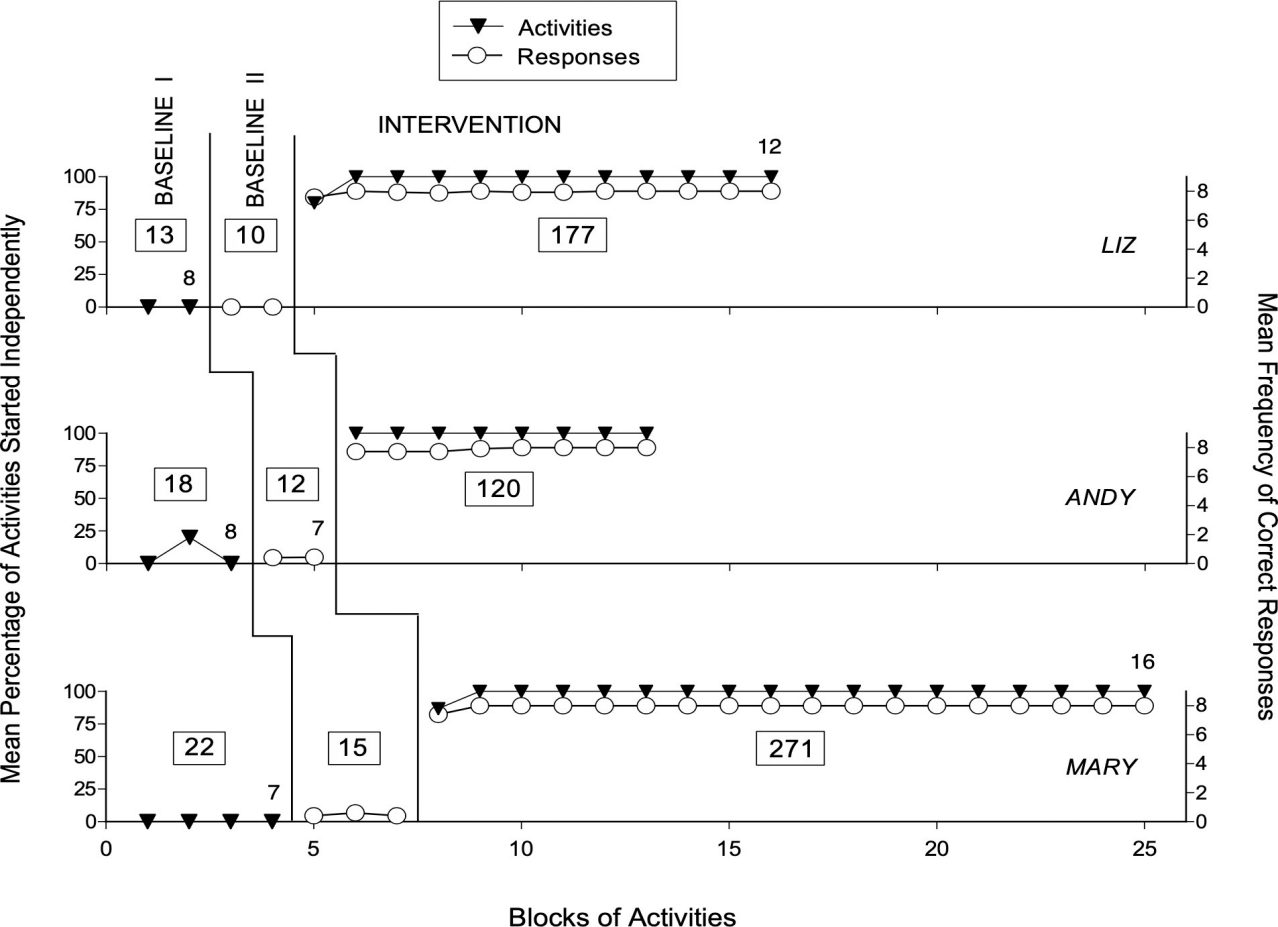
The three panels report the data for Liz, Andy, and Mary, respectively. The black triangles represent the mean percentage of activities started independently over blocks of five activities during Baseline I and 15 activities during the Intervention phase. The open circles represent the mean frequency of correct responses per activity over blocks of five activities during Baseline II and 15 activities during the Intervention phase. A nonstandard block at the end of a baseline phase or the Intervention phase is marked with a number indicating how many activities such block includes. The numbers inside the boxes indicate how many activities were presented to each participant during Baseline I, Baseline II, and the Intervention.

**Fig 2:**
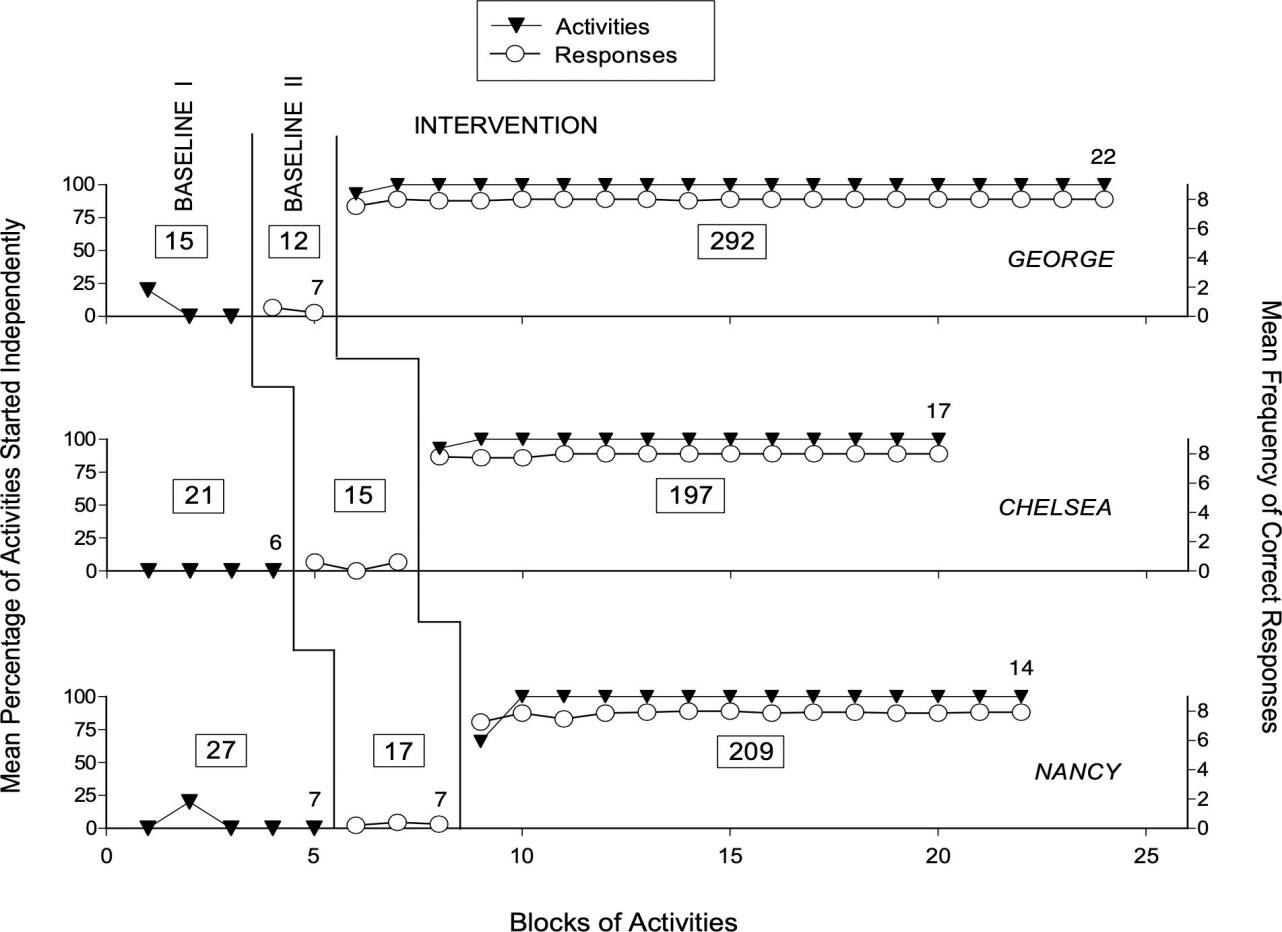
The three panels report the data for George, Chelsea, and Nancy, respectively. Data are plotted as in Fig 1.

Figs 1 and 2 report the baseline and intervention data for the participants using verbal instructions (i.e., Liz, Andy, and Mary) and the participants using pictorial instructions (i.e., George, Chelsea, and Nancy), respectively. The black triangles represent the mean percentage of activities started independently over blocks of five activities during Baseline I and over blocks of 15 activities during the Intervention phase. The open circles represent the mean frequency of correct responses per activity over blocks of five activities during Baseline II and over blocks of 15 activities during the Intervention phase. Any nonstandard block at the end of a phase is marked with a number that indicates how many activities such block includes. The numbers inside the boxes reported in the figures’ panels indicate how many activities in total were presented to each participant during the different phases of the study.

Baseline I included 13 (Liz) to 22 (Mary) activities for the participants relying on verbal instructions (see Fig 1) and 15 (George) to 27 (Nancy) activities for the participants relying on pictorial instructions (see Fig 2). The mean percentage of activities started independently during Baseline I varied between zero and near 7. Andy, George and Nancy were the only participants who started one activity independently, and thus had one data point with a 20% performance level (see Figs 1 and 2). Baseline II included 10 (Liz) to 15 (Mary) activities for the first group of participants (see Fig 1) and 12 (George) to 17 (Nancy) activities for the second group of participants (see Fig 2). The mean frequency of correct responses per activity was below 0.5 **(**out of a maximum possible of 8**)** for all participants (see Figs 1 and 2).

The Intervention phase included 120 (Andy) to 271 (Mary) activities for the first group of participants (see Fig 1) and 197 (Chelsea) to 292 (George) activities for the second group of participants (see Fig 2). Differences in the number of activities used during the Intervention were mainly due to participants’ availability. The mean percentage of activities started independently was virtually 100 for all participants, with exceptions concentrated in the first block of 15 activities (i.e., when the research assistant used prompts/guidance to overcome any participant’s problem or delay in relation to the smartphone’s reminders and instructions) (see Figs 1 and 2). The mean frequency of correct responses varied between 7.8 (Nancy) and virtually 8 (Mary and George) per activity. That is, responses not correct were rare or virtually absent.

Given the obvious difference (i.e., absence of any overlap) between the Baseline data and the Intervention data on these two measures, the use of the Kolmogorov-Smirnov test to evaluate the statistical significance of such difference was deemed unnecessary and thus omitted. The mean amount of time required for carrying out an activity during the Intervention phase ranged between about 6 min (Liz) and 10 min (George).

## Discussion

The data from the Intervention phase indicate that all participants were successful in using the technology system to independently start and carry out various activities at different times of the day. These data support previous findings in the area suggesting that technology-aided programs can be effectively used for supporting participants with intellectual and sensory or sensory and motor disabilities (a) in their performance of functional activities [6,13,20,30] and (b) in their determination to start the activities on their own at the appropriate times [15,21]. The study also confirms that a relatively simple technology system may be set up to provide instructions functionally connected to the participants’ responding [16,22]. The same technology system may be designed to allow and facilitate the scheduling of flexible activities (i.e., activities that can change over different days, in line with environmental requirements and participant’s conditions). In view of the results reported, a number of considerations may be in order.

First, the very high frequency of correct activity responses produced by the participants may be attributed (a) in part to the fact that the responses were familiar to the participants, and (b) in part to the fact that the instructions were functionally connected to the participants’ responding. Although no specific assessment was carried out in this study as to the impact of using instructions functionally connected to responding as opposed to instructions presented at pre-arranged time intervals (i.e., independent of responding), two points suggest that such an impact was relevant. The first point is the data reported by Lancioni et al. [22] showing that participants with intellectual and other disabilities tended to have a significantly more accurate performance with the former type of instructions (i.e., functionally connected to the participants’ responding) than with the latter type of instructions (i.e., presented at pre-arranged intervals). The second point is the data of previous studies using instructions presented at pre-arranged intervals [e.g., 14,15,20,21]. Those data only rarely showed levels of virtually 100% correct responding.

Second, performing activities with high accuracy is likely to make the participants feel at ease during the engagement and perceive the technology system as friendly. Both these aspects would seem relevant to increase the participants’ quality of engagement and eventually their satisfaction with the engagement, with positive implications for their mood and general quality of life [31,32]. Starting the activities independent of staff input (and at the appropriate times) could increase the participants’ sense of control over their occupational situation and improve their social image as well as the level of approval they may receive within the daily context [16,22,33].

Third, the possibility of arranging the daily activities in a flexible manner (by selecting the responses to be included so that the activities can better match environmental requirements and participants’ conditions) may be practically relevant [34–36]. In fact, it may give staff the freedom and opportunities to set up the best occupational schedule for the single participants on a daily basis, with minimal time investment. Obviously, this possibility does not exclude that some of the activities (or all of the activities for some participants) may remain unchanged over any period of time.

Fourth, the technology system used in this study may be viewed as a relatively simple tool that is suitable for participants relying on verbal instructions as well as participants relying on pictorial instructions. The system, moreover, could be improved over time, based on additional participants’ data and staff feedback [37–40]. The fact that the system relies on fairly affordable everyday commercial components can make it accessible to rehabilitation and care contexts. Its present cost may be estimated at about US $550 (i.e., approximately US $175 for the Samsung smartphone, US $200 for the four Philips Hue sensors, US $25 for the mini speaker, and US $150 for the Philips Hue Bridge, the Philips Hue smart bulb, and the 4G Long-Term Evolution Wi-Fi router). A possible obstacle in accessing and using such a system on an immediate basis may be represented by the fact that it is not a ready-made (off-the-shelf) tool but rather a tool that needs to be arranged through the aforementioned commercial components and programmed for the intervention purpose.

### Limitations and future research

The study presents three basic limitations, that is, the small number of participants and lack of follow-up data, the absence of an evaluation of participants’ satisfaction with the use of the system, and the absence of an evaluation of staff opinion about the system. The first limitation, which does not at this time allow one to make general statements about the overall potential and usability of the technology system reported, may be addressed through replication studies involving follow-up checks. These studies could help determine the reliability of the system and, possibly, of upgraded versions of it in promoting and maintaining independent activity performance of people with intellectual and multiple disabilities [41–43].

The second limitation (i.e., lack of data on participants’ satisfaction about the system) might be addressed by (a) asking the participants to choose between activities to carry out with the support of the system and other daily activities to be performed with some staff support [44], and (b) observing the participants’ behavior during the two activity situations and determining possible differences in expressions of positive mood (e.g., smiles) during their engagement [45–47]. The third limitation (i.e., lack of data on staff view about the system) might be addressed by carrying out a staff survey. The survey could be arranged through a brief questionnaire (concerning the efficacy, friendliness, and applicability of the system) that staff persons would be asked to complete after they have watched a brief video illustrating the functioning of the system [48–50].

In conclusion, the results suggest that the technology system was highly effective in helping people with intellectual and sensory or sensory and motor disabilities to start and carry out functional activities independently. The system also allowed to easily change the activities scheduled from day to day, based on environmental requirements or participants’ conditions. While the results are encouraging, no definite statements can be made about the system’s overall potential and usability until new research has addressed the limitations of this study. New research may also try to further develop the present system to improve its effectiveness and extend its usability across participants with different characteristics.
